# Application of the Photoacoustic Approach in the Characterization of Nanostructured Materials

**DOI:** 10.3390/nano12040708

**Published:** 2022-02-21

**Authors:** Mykola Isaiev, Gauhar Mussabek, Pavlo Lishchuk, Kateryna Dubyk, Nazym Zhylkybayeva, Gulmira Yar-Mukhamedova, David Lacroix, Vladimir Lysenko

**Affiliations:** 1 Université de Lorraine, CNRS, LEMTA, 54000 Nancy, France; mykola.isaiev@univ-lorraine.fr (M.I.); david.lacroix@univ-lorraine.fr (D.L.); 2 Institute of Experimental and Theoretical Physics, Al-Farabi Kazakh National University, 71, Al-Farabi Ave., Almaty 050040, Kazakhstan; naz-30@mail.ru (N.Z.); gulmira.yar-muhamedova@kaznu.kz (G.Y.-M.); 3 Institute of Information and Computational Technologies, 125, Pushkin Str., Almaty 050000, Kazakhstan; 4 Institute of Engineering Physics for Biomedicine, Laboratory “Bionanophotonics”, National Research Nuclear University “MEPhI”, 115409 Moscow, Russia; vladimir.lysenko@univ-lyon1.fr; 5 Faculty of Physics, Taras Shevchenko National University of Kyiv, 64/13, Volodymyrska Str., 01601 Kyiv, Ukraine; pavel.lishchuk@knu.ua (P.L.); kateryna.dubyk@gmail.com (K.D.); 6 Light Matter Institute, UMR-5306, Claude Bernard University of Lyon/CNRS, Université de Lyon, 69622 Villeurbanne, France

**Keywords:** photoacoustics, silicon nanostructures, porous nanomaterials, thermal properties, photohyperthermia

## Abstract

A new generation of sensors can be engineered based on the sensing of several markers to satisfy the conditions of the multimodal detection principle. From this point of view, photoacoustic-based sensing approaches are essential. The photoacoustic effect relies on the generation of light-induced deformation (pressure) perturbations in media, which is essential for sensing applications since the photoacoustic response is formed due to a contrast in the optical, thermal, and acoustical properties. It is also particularly important to mention that photoacoustic light-based approaches are flexible enough for the measurement of thermal/elastic parameters. Moreover, the photoacoustic approach can be used for imaging and visualization in material research and biomedical applications. The advantages of photoacoustic devices are their compact sizes and the possibility of on-site measurements, enabling the online monitoring of material parameters. The latter has significance for the development of various sensing applications, including biomedical ones, such as monitoring of the biodistribution of biomolecules. To extend sensing abilities and to find reliable measurement conditions, one needs to clearly understand all the phenomena taking place during energy transformation during photoacoustic signal formation. Therefore, the current paper is devoted to an overview of the main measurement principles used in the photoacoustic setup configurations, with a special focus on the key physical parameters.

## 1. Introduction

**Figure 1:**
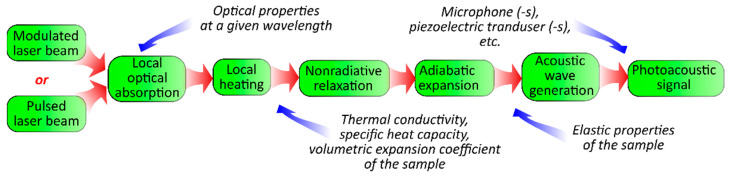
Physical processes involved in PA signal formation.

The photoacoustic (PA) effect is the generation of pressure perturbations in a medium due to its heating with non-stationary electromagnetic radiation. One of the main mechanisms responsible for the effect is the heating of the medium because of the non-radiation relaxation of elementary excitations (electrons, phonons, etc.). The relaxations arise due to the absorption of the radiation, and the subsequent thermal expansion leads to the formation of an acoustical vibration in the materials (see Figure 1) [1]. Thus, the PA response is sensitive to the optical, thermal, and elastic properties of the medium. Moreover, PA methods are a powerful group of approaches for the sensing of various material properties. These can be applied for the investigation of systems in gas, liquid, and solid phases [2,3,4,5].

The manuscript is organized as follows:

- Section 1 (this section) provides a brief introduction to the history and physical background of the photoacoustic effect.
- Section 2 is devoted to the gas–microphone photoacoustic technique. In this, the first section describes the background, status, and future prospects of the approach. The second section is devoted to the mathematical basis that allows us to describe the signal formation. The third section provides insight into the main configurations of the gas–microphone photoacoustic cells. The last section provides examples of the application of the photoacoustic approaches in the evaluation of thermal and optical properties of nanostructured materials.
- Section 3 reports the features of photoacoustic signal formation by implementing the PA technique with the configuration of piezoelectric detection. The first section gives a short overview of the theoretical basis for the description of the signal’s formation in this case, and the second section gives some examples of the application of the piezoelectric approaches in the characterization of nanomaterials.
- Section 4 provides a short description of the application of the photoacoustic methods in imaging (the first section) and visualization (the second section) with respect to biomedical and materials research applications.
- Section 5 provides an overview of the use of the photoacoustic approach for FT-IR spectroscopy with a gas–microphone configuration (the first subsection) and its application in the nanosystem investigation in different phases.

The PA effect was discovered and reported by A.G. Bell at the end of the 19th century. However, at that time, the absence of a reliable light source for heating did not allow further development of the PA-based approaches. The situation changed with the discovery of lasers. Their use in PA methods has simplified the process of non-destructive probing of various material properties.

It should be noted that the multiparametric sensitivity of the PA response, on the one hand, provides the possibility of studying a variety of parameters simultaneously and, on the other hand, complicates the analysis of experimental data. Thus, the understanding of the mechanisms that occur during energy transformation, i.e., electromagnetic radiation to the acoustical oscillations, is necessary to perform reliable measurements of the desired parameter. It offers the possibility of developing specific PA configurations with maximum sensitivity for the chosen parameters and the minimum possible sensitivity for the others. As an example, the PA gas–microphone system with opened/closed window configurations can be mentioned here. Although one is sensitive to the mechanical stresses arising in the system, another is more reliable for measurements of thermal properties.

Thus, PA methods are powerful tools for the investigation of a wide variety of properties, which is essential in various areas of material research and biomedical applications. The main advantages of such approaches are that they are contactless and they offer the possibility of extracting information non-destructively. The sensitivity of the signal to several parameters provides the possibility of developing a PA configuration for imaging the heterogeneities in materials with high contrast. Moreover, a significant advantage of the approach is the possibility of performing in situ measurements [6].

At the moment, several reviews have been published in which the possibility of using PA methods is partially or completely considered. For example, in [7], a comparative review of non-destructive thermal methods of the diagnostics of material properties is carried out. In this review, PA methods are briefly compared with other non-stationary photothermal methods. In the review of [8], attention is focused on the PA effect as one of the main effects in the usage of laser photothermal spectroscopy. Other reviews have focused on specific areas of the application of PA methods. For example, photoacoustic tomography (PAT) methods are considered a powerful tool in preclinical and clinical visualization [9], for gas environment research [2], for the characterization of solid structures [5], etc.

The aim of this article is to provide an overview of the commonly used PA techniques by demonstrating the sensitivity of such methods to specific parameters. We will start with the classic gas–microphone and piezoelectric PA techniques and then study the applications of both approaches in sensing the properties of different media. Special attention will be paid to the application of PA approaches in imaging and infrared (IR) spectroscopy.

## 2. Photoacoustic Gas–Microphone Technique

### 2.1. PA Gas–Microphone Technique: Background, Status, and Future Prospects

This section focuses on the most-used PA technique for thermal, optical, and mechanical property evaluation in solid materials, namely the PA gas–microphone method. 

It has been demonstrated that the PA technique with gas–microphone (GM) recording is a valuable method with low-cost sources for the non-destructive study of matter in different aggregate states [7]. The main advantages of this method are: (a) its versatility while creating a suitable system for the fast diagnosis of different samples with variable degrees of sensitivity and (b) its ability to analyze parameters along with the depth profile of the studied structures.

The optical excitations used with this technique are supplied periodically, resulting in periodic temperature changes in the sample, with a subsequent indirect generation of informative sound waves in the adjacent isolated gas that are registered by the microphone. The resulting PA signal is sensitive to the structural properties of materials, i.e., it depends on the sample’s absorbing properties and heat diffusion properties. The modulation of thermal perturbations inside the structure helps to overcome drawbacks associated with the contact and steady-state diagnostic methods, such as thermal contact resistance and parasitic heat losses [10].

During the last three decades, the crucially reduced heat transport in low-dimensional semiconductor structures from group IV (silicon, germanium, etc.) in comparison to the bulk materials has been reported in numerous studies [11,12,13,14]. These features, combined with new fabrication techniques, make these materials attractive for implementation in various applications. For example, the low thermal conductivity of nanostructured silicon due to spatial confinement makes it an excellent thermal insulator for MEMs and NEMs. Moreover, its thermal and optical functionalities are good enough for its use as an efficient structural component of solar cells, thermoelectric cells, and sensor devices and systems.

The synthesis of porous semiconductor materials is possible and convenient for the miniaturization of structural elements of various functional systems with the desired distribution of heat transfer properties. Among the main methods to obtain such nanostructures, one can use controlled electrochemical and metal-assisted chemical etching, which makes it possible to form a porous fractal or a well-ordered monolayer, a multilayer stack with different porosities bonded on a bulk substrate, or even free-standing structures [15,16]. The thickness of the resulting structures can be tuned to range from several microns to hundreds of microns, and the porosity can also vary over a wide percentage range. Notably, the properties of the obtained nanomaterials significantly depend on (1) the properties of the starting material and (2) the fabrication parameters of the structure. However, at the atomic scale, these processes are stochastic in nature and, even now, the exact mechanism of pore formation is not fully defined.

The development of the new sensing approaches [17] for the detection of the pressure’s perturbation in gas–microphone PA cells opens a new perspective on the application of the approach in PA-IR spectroscopy and in biomedical devices. Improving the sensitivity of the detectors creates the possibility for further miniaturization of the PA cells and increasing the range of its applications for the study of nanostructured materials [2]. 

Thus, it is necessary to emphasize the importance of the PA methods applied for the analysis and further development of low-dimensional semiconductor materials. Our main goal is to analyze the experimental setup proposed for the evaluation of the properties of nanostructured porous semiconductor materials. Another purpose of this section is to briefly detail the models of heat transport in such materials by using the PA technique.

### 2.2. Main Concepts of PA Signal Analysis Obtained by the Gas–Microphone Technique

The photoacoustic GM technique is one of the most widespread low-frequency methods for PA signal detection. The PA response formation in the gas–microphone cell occurs through several mechanisms. Generally, the main mechanism for front- and rear-surface illumination configurations is called a gas–piston model (see Section 2.3 and Section 2.4). According to this model, the photoinduced heat in the studied sample enables subsequent non-radiative de-excitation–relaxation processes followed by the generation of a fast-decaying thermal wave. The wave propagates over the interface into the adjacent isolated gas inside the PA cell. Then, the thermal wave decays in a thin layer of gas adjacent to the sample, causing the periodic temperature fluctuations matching the modulation of laser irradiation. Therefore, the periodical heating of the gas causing its expansion and contraction leads to compressions and dilations in the rest of the gas in the PA cell, detected by the in-built microphone as a PA signal. A characteristic feature of low-frequency photoacoustic methods is the evaluation of thermophysical and optical properties of the samples based on the amplitude–frequency (AFC) and phase–frequency (PFC) analysis of the pressure oscillations.

Let us describe the temperature wave formation in the sample under irradiation with periodically modulated radiation by using the front-surface illumination configuration in a one-dimensional approximation. The problem in the 3D case shows that its reduction to a one-dimensional approximation works well when the excitation source’s radius is much larger than the thermal diffusion length.

Let us consider the case when all light radiation is absorbed and thermalized in the sample by radiation-free relaxation mechanisms. When the total intensity (*I*_0_) of the excitation irradiation is modulated by the sinusoidal law, its time dependence can be given in the form *I*_0_ (1 *+ cos*(*ωt*))/2, where ω is the angular frequency of modulation of the excitation irradiation. If the coefficient of optical absorption in the test sample is denoted as α, the heat source power density due to the absorption of radiation in the sample can be described as follows:

(1) $$P\left(x,t\right)=\frac{1}{2}I_{0}\alpha exp\left(\alpha x\right)\left(1+cos\left(\omega t\right)\right)$$

In this case, the variable component of thermal perturbation in the sample can be described in a complex form based on the heat equation:

(2) $$c_{s}\rho_{s}\frac{\partial T_{s}^{AC}}{\partial t}=\chi_{s}\frac{\partial^{2}T_{s}^{AC}}{\partial x^{2}}+\frac{1}{2}I_{0}\alpha Re(\exp\left(i\omega t\right))$$

where *c_s_*, *ρ_s_*, and *χ_s_* are the specific heat, density, and thermal conductivity of the tested sample, respectively. The index “s” means that the corresponding quantity is related to the sample, and the superscript “AC” of the temperature indicates that the solution of this equation is the result of the action of the variable component of the excitation radiation.

The traditional approach used to describe physical systems that are subject to an external harmonic excitation is to consider the system in the complex plane:

(3) $$c_{s}\rho_{s}\frac{\partial \Theta_{s}}{\partial t}=\chi_{s}\frac{\partial^{2}\Theta_{s}}{\partial x^{2}}+\frac{1}{2}I_{0}\alpha \;exp\left(\alpha x\right)\exp\left(i\omega t\right)$$

where Θ is an analytical extension of $T^{\mathrm{AC}}$ to the complex plane $\left(T^{\mathrm{AC}}=Re\left(\Theta \right)\right)$. 

Thus, the solution of the equation can be represented as:

(4) $$T\left(z,t\right)=T_{0}+T^{\mathrm{DC}}+Re\left(\Theta \left(z,t\right)\right)$$

where *T*_0_ is the initial temperature of the test sample and the environment inside the PA cell and $T^{\mathrm{DC}}$ is the steady-state average temperature of the sample’s surface during its irradiation.

To obtain the spatial distribution of the variable component of the thermal perturbation θ(x), the substitution Θ(x,t)=θ(x)exp(iωt) is provided, and after simplifications of Equation (3), the following equation can be obtained:

(5) $$\frac{\partial^{2}\theta_{s}}{\partial x^{2}}-\frac{i\omega }{D_{s}}\theta_{s}=-\frac{1}{2}\;\frac{I_{0}\alpha }{D_{s}}\exp(\alpha x)$$

where *D_s_* = *χ_s_*/*c_s_ ρ_s_* is the thermal diffusivity coefficient of the sample.

The corresponding equations can also be obtained for the spatial distribution of the variable component of the temperature perturbation in the gas, which practically does not absorb the excitatory radiation (*θ_g_*), and in the substrate material (*θ_b_*):

(6) $$\{\begin{array}{c} \frac{\partial^{2}\theta_{g}}{\partial x^{2}}-\frac{i\omega }{D_{g}}\theta_{g}=0\\ \frac{\partial^{2}\theta_{b}}{\partial x^{2}}-\frac{i\omega }{D_{b}}\theta_{b}=0 \end{array},$$

where *D_g_* and *D_b_* are the thermal diffusivity coefficients of the gas and the substrate, respectively.

Thus, the spatial distribution of temperature perturbation in the PA cell can be represented as follows:

(7) $$\theta \left(x\right)=\left\{ \begin{array}{cc} \theta_{g}\left(x\right)=Aexp\left(-\sigma_{g}x\right) & \;\;l_{g}>x>0\\ \theta_{s}\left(x\right)=Bexp\left(-\sigma_{s}x\right)+Cexp\left(\sigma_{s}x\right)-L\;exp & \;\;-l_{s}<x<0\\ \theta_{b}\left(x\right)=Eexp\left(\sigma_{b}x\right) & -\left(l_{s}+l_{b}\right)<x<-l_{s} \end{array}\right.$$

where *A*, *B*, C, and *E* are the complex constants that can be determined from the boundary and interface conditions; *l_g_*, *l_s_*, and *l_b_* are the thickness of the gas, sample, and substrate material layers, respectively; *l_g_ >> l_s_*, *l_b_ >> l_s_*; with $\sigma_{j}=\left(1+i\right)\sqrt{\omega /\left(2D_{j}\right)}$, where index *j* refers to the gas, sample, and substrate material layers, respectively; and $L=I_{0}\alpha /2\chi_{s}\left(\alpha^{2}-\sigma_{s}^{2}\right).$

**Figure 2:**
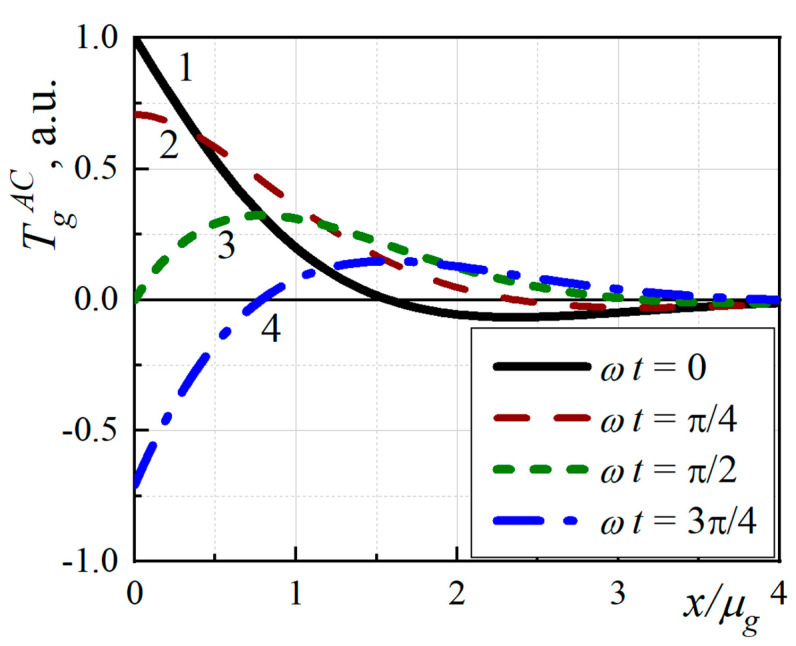
Spatial distribution of the variable component of temperature perturbation $\left(T_{g}^{AC}\right)$ in the gas layer adjacent to the sample’s surface: *ωt =* 0 (1), *ωt = π/*4 (2), *ωt = π/*2 (3), and *ωt =* 3*π/*4 (4).

Since the gas inside the PA cell does not absorb the excitation radiation, the presence of nonequilibrium temperature perturbation (*θ_g_*) is associated with its heating as a result of the presence of heat flux from the surface of the studied structure. Therefore, the parameters of such perturbations will depend only on the thermophysical characteristics of the sample. Let us analyze the spatial distribution of the variable component of temperature in the gas layer adjacent to the sample. Figure 2 shows that this distribution exponentially decreases with time while reaching the thermal diffusion length (μ_g_ = 1/Re(σ_g_)) and then oscillates with a period of *2π/*Im(*σ_g_*).

Because of the oscillating nature of the variable component of the thermal perturbation, it can be considered a highly damping system and it may be considered in the framework of “thermal wave approach” formalism. This is because the variable component of the perturbation behaves as a fast-extinguishing wave that has a length proportional to the characteristic depth of attenuation (Re(*σ_g_*) *=* Im(*σ_g_*)).

A thin layer of gas, while expanding, leads to pressure variation inside the PA cell. The displacement (*δ_x_*) of the gas piston can be represented as follows:

(8) $$\delta x=\frac{1}{T_{0}}\int_{0}^{2\pi {µ}_{g}}T^{AC}\left(x\right)dx$$

Since the heated gas layer is much thinner than the total thickness of the gas in the PA cell, the formation of the acoustic response can be described in the adiabatic approximation. Using the state equation for such a process (perfect gas, $PV^{\gamma \;}$= constant), the pressure changes inside the PA cell can be represented as:

(9) $$\delta P\left(t\right)=\frac{\gamma P_{0}}{l_{g}T_{0}}\int_{0}^{2\pi {µ}_{g}}T^{AC}\left(x,t\right)dx$$

where *P*_0_ and *V*_0_ are the initial pressure and the inner space volume of the PA cell, respectively.

Equation (9) describes the time dependence of the pressure variations inside the PA cell. Since experimental studies are based on the amplitude and phase shift of the PA signal and its frequency characteristics, the following expression is used to describe them:

(10) $$\delta \tilde{P}\left(\omega \right)=\frac{\gamma P_{0}}{l_{g}}\delta \tilde{x}=\left(\frac{\gamma P_{0}}{l_{g}T_{0}}\int_{0}^{2\pi {µ}_{g}}\theta_{g}\left(x,\omega \right)dx\right)exp\left(i\omega t\right)=A_{p}exp\left(i\omega t\right)$$

where $\delta \tilde{P}$ and $\delta \tilde{x}$ are the analytic continuation of δP(t) and δx in the complex plane, respectively and *A_p_* is a complex amplitude that contains information about the amplitude and phase shift of the PA signal relative to the reference signal.

#### 2.2.1. Thermal Diffusivity Measurements

The critical frequency method is considered a classic method to evaluate the thermophysical parameters of a sample when front-surface illumination is used [18]. This method is based on the thermal wave approach. The value of the thermal diffusivity (*D*) of the structure is obtained by taking into account the value of the modulation frequency (*f_c_*)*,* for which the length of the thermal diffusion *µ* of the sample coincides with its size *d* (*µ = d*). Information about the position of this frequency is contained in the analysis of the AFC of the PA response on a double logarithmic scale. In the latter, a bend divides the graph into two areas. Each area corresponds to a distinct case, i.e., when the sample is “thermally thin” or “thermally thick”. 

Essential for this method’s implementation is to have a significant difference in the thermal properties of the sample layer and the backing material. If the value of *f_c_* is known, the thermal diffusivity is determined by the following equation:

(11) $$D=\pi f_{c}d^{2}.$$

In the case of a two-layer structure, the upper layer, with length (*l*), has a low thermal diffusivity compared to the lower layer and the position *f_c_* is determined from the analysis of AFC, where visually there is a change in the slope of the signal’s amplitude from $\left(\sim f^{-0.5}\right)$ to (~$f^{-1}$). At this bending frequency, the thermally thin top layer becomes thermally thick. When the specific heat (*c*) and the density (*ρ*) of the top layer are known (taking into account the porosity of the sample), this method, using the well-known equality D=χ/(ρc), enables the calculation of its thermal conductivity (*χ*):

(12) $$\chi =c\rho \pi fl^{2}.$$

In particular, this case relates to structures containing a porous layer on a bulk semiconductor substrate [19]. In the latter study, the PA method appears as a powerful tool to investigate the morphological properties of a porous silicon structure by taking into account the dependence of the thermal conductivity of the considered mesoporous silicon layer on its porosity. The thermal conductive increase in a composite system in which the original structures are made of porous silicon has shown that the oil-filled pores can be used as a marker for the morphology of the porous material.

The second configuration of the experiment (the irradiation of the rear surface of the sample) is often applied to analyze the phase shift of the signal relative to the phase of modulated light. This parameter mainly depends on the characteristics of the thermal wave through the structure and contains information about its thermal diffusivity.

This configuration is more suitable for the study of both homogeneous and two-layer samples when the thermal properties of one layer is known. When considering the case of a thermally thin sample, the problem is reduced to the calculation of the effective thermal diffusion of the structure within the framework of the thermal resistance analogy model [20]. When the two-layer sample is thermally thick and has an equal thermal contribution of both layers, it is possible to use the approximation of a homogeneous medium with effective thermal diffusivity (*D_eff_*) [21]:

(13) $$\frac{l_{1}+l_{2}}{\sqrt{D_{eff}}}=\frac{l_{1}}{\sqrt{D_{1}}}+\frac{l_{2}}{\sqrt{D_{2}}}$$

where each index indicates that the parameter belongs to the first or the second layer of the system.

When studying a two-layer semiconductor system, which consists of a porous layer and a thermally thin monocrystalline layer in the operating frequency range, one can use the approximation of a phase shift that is typical for illuminating an effective homogeneous layer of a sample with a thickness *l*. If the experimental results are normalized for the analysis of a reference sample (single-crystal silicon layer), the resulting phase shift will be the result of a time delay in the thermal perturbation passing through the porous silicon layer. The PA response phase in the case of $f>D/l^{2}$ will be dependent on the thermal diffusivity of the layer as follows:

(14) $$\Delta \varphi \left(f\right)=-\pi /4-l/{\left(D/\pi f\right)}^{1/2},$$

Thus, the thermal diffusivity with the subsequent thermal conductivity values of the porous layers can be determined from the slope of the fitting line of normalized PFC. These investigations were developed for the comparative evaluation of the thermal conductivity of porous silicon with and without liquid in pores to study the morphological features of a porous nanostructure [22].

#### 2.2.2. Characterization of Low-Dimensional Semiconductor Materials by Variable Temperature Component Distribution Modeling

To deduce the thermal conductivity values of the porous layer from the two-layered structure under consideration, the measured experimental AFC and PFC can be treated through the simulation of temperature field *(θ)* distribution with the use of the Rosencsweig–Gersho approach (RG model) [23]. A prerequisite to using this model for the analysis of the PA response is the difference between the thermophysical and optical properties of the two layers of the structure.

**Figure 3:**
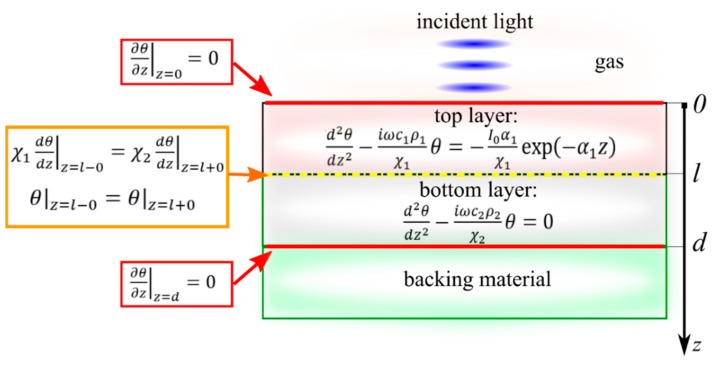
Cross-sectional view of the model characterized by heat conduction equations for each layer with the corresponding boundary conditions. The location of the layer under investigation depends on the configuration of the PA cell; the top layer is assigned in the case of front-surface illumination configuration, and the bottom layer is assigned in the case of rear-surface illumination configuration; α is the optical absorption coefficient, and ω is a cycling frequency.

It should be noted that in the framework of this model, the porous layer of the structure is considered a homogeneous material with its own effective value of thermal conductivity. This approximation can be used because in the considered frequency range of experiments, the thermal diffusion length in the layer is several orders of magnitude higher than the characteristic pore size of the typical porous Si or Ge matrix. Thus, the problem is reduced to finding the solution of a 1D heat conduction equation with boundary conditions (see Figure 3).

The following must be considered for the boundary conditions of the proposed two-layer structure:

- The absence of heat outflow at the lower and upper boundaries of the studied structure;
- The continuity conditions for both temperature and heat flow at the interface between the two layers of the system.

When using a PA cell in the front illumination configuration, in the approximation of the strong absorption of light by the sample, the upper layer (see Figure 3) is the layer with the unknown thermal and optical parameters. Thus, according to the RG model, the pressure fluctuations inside the PA cell, in this case, are described as follows:

(15) $$A\left(\omega \right)\;\;\sim \;P\sim \overset{-\infty }{\underset{0}{\int }}\theta \left(0\right)\exp\left(\sqrt{\frac{i\omega c_{g}\rho_{g}}{\chi_{g}}}z\right)dz=-\theta \left(0\right)\sqrt{\frac{\chi_{g}}{i\omega c_{g}\rho_{g}}}$$

where *θ*(0) is a variable temperature component on the surface of the top layer of the system that is adjacent to the isolated gas of the PA cell.

When using a PA cell in the rear illumination configuration, the porous layer is located on the opposite side of the incident light. Thus, the informative response will be provided in the gas that is in contact with a lower layer due to the passage of thermal perturbation through the whole structure. In this case, pressure fluctuations can be represented as follows:

(16) $$A\;\left(\omega \right)\;\sim \;P\;\sim \overset{+\infty }{\underset{d}{\int }}\theta \left(d\right)\exp\left(-\sqrt{\frac{i\omega c_{g}\rho_{g}}{\chi_{g}}}\left(z-d\right)\right)dz=\theta \left(d\right)\sqrt{\frac{\chi_{g}}{i\omega c_{g}\rho_{g}}}$$

where *θ*(*d*) is a variable temperature component on the surface of the bottom layer of the system that is adjacent to the isolated gas of the PA cell.

The evaluation of the results is carried out by varying the parameters of thermal conductivity and optical absorption of the studied porous layer to minimize the difference between theory and experiment.

### 2.3. Experimental Setup of the PA GM Technique

**Figure 4:**
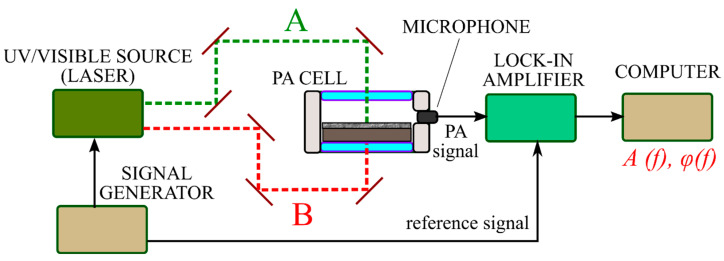
Typical photoacoustic setup for the thermal and optical property characterization of layered semiconductor materials. Case A corresponds to the front-surface illumination configuration; case B refers to the rear-surface illumination configuration.

The experimental setup for the gas-phase measurement is illustrated in Figure 4. Generally, semiconductor materials are characterized by PA gas–microphone measurements in the UV–visible range. A laser with the desired wavelength modulated electrically by a square signal generator or mechanically by a chopper is used as a non-stationary light irradiation source. Alternatively, LEDs can also be used or even broadband radiation sources followed by optical filters. Periodic light radiation is directed and focused on the surface of the sample by using an optical system. It triggers the modulation of the photoinduced heating with subsequent cooling.

Typically, the experiment can be carried out in front- or rear-surface illumination configurations. For both methods, the PA cell is usually a small container for the studied sample with a microphone built into the wall. Here, the microphone can detect the PA signal inside the PA cell in a wide range of radiation frequencies without distortion. When a solid sample is under investigation, the measurement of the generated PA response is carried out in the gas that is in contact with the sample’s surface. It is difficult to analyze a solid material with big thermal conductivity by modulated PA excitation and subsequently detect the directly generated acoustic wave by a microphone. In this case, indirect PA signal detection is employed due to the strong acoustic impedance mismatches between the solid and gas phases. More accurate results are obtained with helium, but the most commonly used gas is air. Eventually, commercial and relatively inexpensive electret or condenser microphones are convenient detectors of the PA response.

For both front- and rear-surface illumination configurations, the PA cell also contains optically transparent glass as one of the walls named the “optical window” of the cell. In the case of front-surface illumination configuration, it plays the role of a wall, which simultaneously performs two functions: (1) the transmission of laser radiation to the sample’s surface inside the cell and (2) the isolation of the internal gas from the external environment. In the other configuration, the window can also act as a damper, blocking the membrane effect [24].

The PA signal detected by the microphone should be compared with the reference signal through modulation by a lock-in amplifier to extract the PA signal’s characteristics. At the same time, it is possible to use either a PC and/or an oscilloscope to evaluate the tightness of the PA cell, analyze the shape of the PA response, or monitor the experimental data obtained in express mode.

For the other walls of the PA cell, it is necessary to use a material that is either optically transparent for the reflected light from the sample or has high reflectivity (>90% for the light wavelength used in the experiment). Of course, the choice of material is also influenced by design factors, for example, the ability of the material to provide internal gas insulation and the ability to be easily processed to provide the desired small dimensions of the internal space of the designed PA cell.

For both illumination configurations, the inner space of the PA cell must be limited since the volume is inversely proportional to the amplitude of the PA response. Generally, when the front-surface illumination configuration is used, the length and width of the sample are in the range from several units to tens of millimeters and the base of the PA cell is reduced close to the corresponding area of the sample. During the experiments, the sample is placed at the bottom of the inner space of the PA cell. The height between the surface of the sample and the optically transparent window of the PA cell must be at least a few millimeters so that the thermal diffusion length of the gas does not reach the window. If the sample is larger in dimensions or is a part of a complete system, then it is possible to attach the upper part of the PA cell with an optical window directly to the study area, provided that the air is securely isolated by the cell above the investigated surface.

Thus, we discussed a situation in which the vibrations of the sample do not impact the formation of the informative response. Experimentally, it can be easily achieved by fixing the sample. In the alternative case, the rear-surface irradiation configuration measurements of the sample can be carried out according to the well-known open-cell PA configuration. It is realized by simply placing the structure directly on the microphone without a PA cell. In this case, if the sample is not rigidly fixed, to ensure gas isolation between it and the microphone, the acoustic waves that will arise in the space between the structure and the microphone will depend on both thermoelastic components and the membrane effect [23]. The open-cell PA method, when the sample is not rigidly fixed, allows for determining the elastic modulus and the coefficient of thermal expansion of the sample [24,25,26]. 

**Figure 5:**
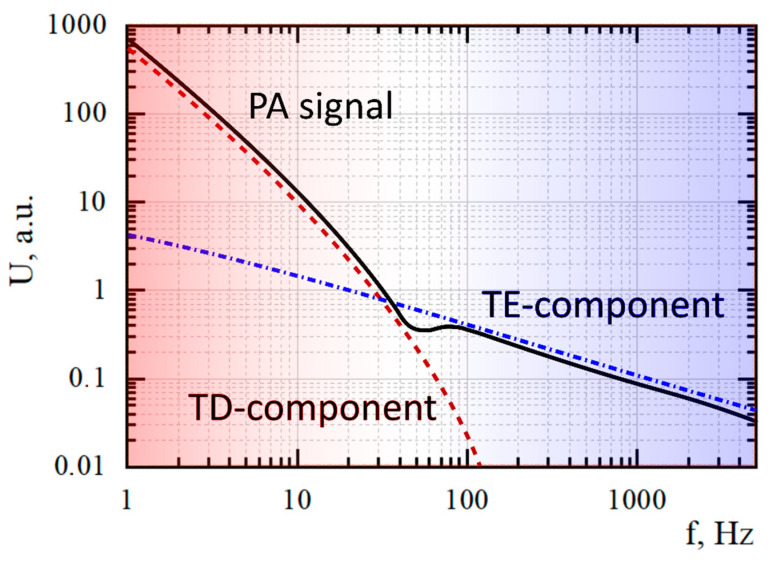
Simulated amplitude–frequency dependencies of the PA signal for the sample based on the open-cell PA configuration. Dashed and dash-dotted lines show the calculated dependencies for the TD component and the TE component of the PA signal, respectively, and the solid line shows the result of summation.

According to this method, the sample is attached to a buffer rubber ring with elastic properties that allow the sample to carry out free thermoelastic deformations during its periodical light irradiation. In this case, the informative PA response is formed by both the gas–piston effect (thermo-diffusion (TD) component of the PA signal that dominates at the low frequencies) and the membrane effect (thermoelastic (TE) component of the PA signal that dominates at the relatively high frequencies) in the internal volume of the PA cell (see the Figure 5). In some cases, the parameters of the sample make a significant contribution to the membrane effect to the PA response in the operating range of modulation frequencies. This, for example, allows for recording the thermoelastic component of the signal both upon the irradiation of porous silicon and a composite system based on it to conduct a complex assessment of their morphological features, as shown in [27].

To summarize, the selection of the most appropriate configurations for a structure’s characterization using the gas–microphone PA technique depends on both the samples’ dimensions and the prediction of a range of properties. Depending on the situation, this can be either an advantage or a disadvantage of this method [7]. It is worth considering the limitations of the PA gas–microphone technique in the working frequency range that can complicate the task of a qualitative analysis of samples due to the possible absence of informative markers on the obtained AFC and PFC of the PA response.

### 2.4. The Application of the Photoacoustic Approaches in the Evaluation of Thermal and Optical Properties of Nanostructured Materials

In most cases, for a rapid analysis of the thermophysical and optical properties of various low-dimensional semiconductor structures synthesized on a bulk substrate, it is enough to use one or several radiation wavelengths as a radiation source. In addition, it should be noted that in any case, PA cells must be calibrated on samples with known properties in order to remove cell effects that may affect the measurement results in experiments.

Hereafter, we describe the methodology for using the proposed PA gas–microphone technique. We use a detailed, accurate, and reliable calculation of the thermal properties of porous semiconductor materials with different morphologies set on a bulk substrate (two-layered structures, where the top layer is under investigation).

This approach has been successfully used to study the thermophysical properties of porous silicon [28,29], porous germanium [30], silicon nanowires [31], and other porous semiconductor materials [32,33,34,35]. Its application allows us to determine the influence of both etching factors and the parameters of the bulk material on the resulting morphology and, as a consequence, on the heat transport properties. In particular, the impact of factors such as porosity, the oxidation degree of porous silicon, the doping level, and the thickness of silicon nanowire arrays or even the impact of filling the porous matrix with liquids on thermal conductivity has been studied.

It should be noted that the PA gas–microphone method requires knowledge of the amount of light absorbed by the sample. Therefore, to qualitatively determine the thermal conductivity of a porous layer, data on the density, the heat capacity, the layer thickness, as well as the optical absorption coefficient at a given wavelength are needed. In some cases, to solve this problem, additional studies of samples are proposed, for example, using ellipsometry [29,30]. However, the ellipsometry method is inapplicable if the sample’s surface strongly scatters light. In this case, it is possible to implement a number of PA configurations for more accurate results (as shown for the samples with high reflectivity, such as silicon nanowires) [31]. A solution to deal with the uncertainty of several parameters of the considered samples was found by using (a) several light sources with different radiation wavelengths and (b) several PA cell configurations. 

Varying the wavelength of the incident radiation induces a variation in the optical absorption coefficient of the material. Using different wavelengths for the two-layered system may cause a change in the informative PA response since, for wavelengths close to ultraviolet light, the sample is typically optically opaque; for wavelengths close to infrared light, the sample is typically optically transparent.

Contrary to the traditional front-surface configuration, a bulk silicon substrate is illuminated in the rear-surface illumination configuration. Therefore, several experimental factors, such as the surface inhomogeneity of nanowires, light-trapping, and other phenomena, that may affect the signal noise can be neglected. This approach makes it possible to have enough data for optical absorption and thermal conductivity parameter evaluation. Moreover, it offers the possibility of studying the optical and thermal properties of porous silicon in situ in the process of its fabrication [36,37,38].

#### 2.4.1. Features of PA Response Formation in Porous Multilayered Structures

The study of thermal transport in multilayer porous (MLP) structures with a spatial distribution of ultrathin layers that differ by porosity is complicated by many factors. In this case, the thermal and optical features of the MLP structure can affect the measurement’s accuracy if using several excitation wavelengths. Among the drawbacks that may significantly complexify measurement analysis while using PA gas–microphone methods in front-surface illumination configuration, the most influential are the thermal boundary and multiple light reflections between the layers within the MLP structure. Thus, the analysis of the experimental measurements requires a more elaborated model of the PA response formation in an MLP semiconductor system, where each layer is characterized by its own thermal and optical properties.

The accurate simulation of the PA response’s formation from a silicon-based MPL structure with a periodic distribution of porosity was proposed in [32]. The analysis of the PA response from the MLP sample was performed during its periodical illumination by a UV light source. In this case, the UV light localizes a heat source in the surface layer of the MLP structure due to its strong light absorption. Moreover, the complex dielectric permittivity calculation of each porous layer was carried out with Bruggeman’s approximation to overcome uncertainty related to the optical parameters of the entire MLP structure. This approach allowed us to simulate a multilayer structure in the effective medium approximation and to determine its thermal conductivity value.

Later, this approach was generalized to cases of the electromagnetic irradiation of samples at different wavelengths [34]. According to the published results, the procedure for analyzing the PA response’s formation from MLP samples involves the following steps:

- Calculation of the complex dielectric permittivity using Bruggeman’s approximation;
- Numerical evaluation of the volumetric heat source from Maxwell equation’s resolution;
- Calculation of the temperature profiles of the photoexcited sample and the fitting procedure with the experimentally obtained PA response using the developed model’s calculations.

The thermal conductivity values of multilayer structures obtained by both approaches are qualitatively correlated and are significantly lower than those estimated in the framework of an electrothermal analogy model for MLP structures. 

The authors demonstrated that the origin of such discrepancies is systematic and related to the presence of thermal boundary resistance between the porous layers of the MLP structure. This approach can be implemented in PA response investigations of other multilayer systems to analyze its thermal transport.

#### 2.4.2. Comparative Magnitude Analysis of the Thin, Porous Films Using the PA Technique

Generally, the working region of the modulation frequencies in PA GM methods is limited due to instrumental effects, PA cell effects, and other factors. In addition, if the typical features of PA response (for example, the bending of AF dependencies) are absent, the evaluation of thermal physical properties may be tricky. Specifically, this concerns the study of thermally thin layers with thicknesses lower than the thermal diffusion length.

**Figure 6:**
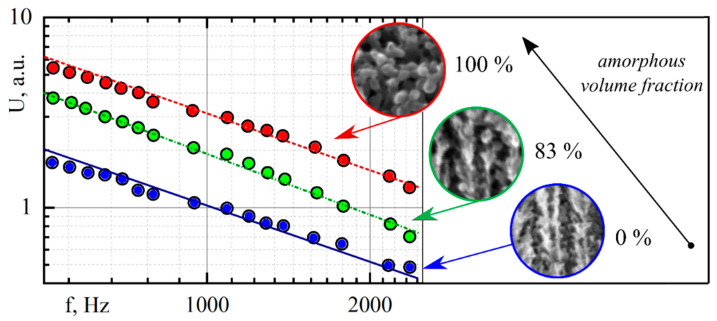
The amplitude–frequency dependencies of the PA signal for the porous silicon samples with 75% porosity and amorphous fractions of 0%, 83%, and 100%. The insets show the 200 nm region of the cross-sectional views of the porous silicon layer, obtained by SEM.

For these kinds of samples, the amplitude of the PA signal versus the frequency has a constant slope across the entire experimental frequency range. However, we can apply the comparative analysis of the magnitude of the PA response if the task is to analyze the impact of various modifications on the thermophysical properties of the thin samples that are similar in their optical properties. For example, the PA GM technique was applied for the comparative thermal conductivity study of partially amorphous porous silicon [39]. The amorphization of porous silicon samples was provided by the irradiation of the porous silicon layer on a c-Si substrate by swift, heavy U ions with adjustable fluence [39,40,41]. The typical AFC of the PA signal obtained for 10 µm porous silicon samples with 75% porosity and a different volume fraction of the amorphous phase is shown in Figure 6.

Considering the strong optical absorption of the samples, the pressure variations inside the PA cell during the measurements can be expressed as follows:

(17) $$A\left(\omega \right)\sim \overset{\infty }{\underset{0}{\int }}\theta \left(z,\omega \right)dz=-\frac{l_{por}}{\chi_{por}}\sqrt{\frac{\chi_{g}}{i\omega c_{g}\rho_{g}}}.$$

where indices “*g*” and ”*por*” indicate that the parameter belongs to the gas or the porous layer, respectively.

In the framework of the following model’s approximations, as one can see from Equation (17), the magnitude of the PA’s signal is inversely proportional to the thermal conductivity of the porous layer. Therefore, the PA GM method allows us to trace the evolution of changes in the thermophysical properties of porous silicon due to its amorphization. Moreover, the specific values of thermal conductivity of the irradiated samples can be determined by using a known value of thermal conductivity of original (non-irradiated) samples of porous silicon:

(18) $$\frac{p^{\left(NI\right)}\left(\omega \right)}{p^{\left(I\right)}\left(\omega \right)}=\frac{\chi_{por}^{\left(I\right)}}{\chi_{por}^{\left(NI\right)}}.$$

where indices “*I*” and “*NI*” denote the dependence of the parameter on the irradiated and non-irradiated porous silicon sample, respectively.

Thus, the decrease in the thermal conductivity of the amorphized porous silicon with the increase in the amorphous phase fraction has been experimentally shown. These results are qualitatively correlated with those that were previously established using the micro-Raman and scanning thermal microscopy methods. Moreover, it was shown that the PA GM method allows us to determine the thermal conductivity of samples with both high porosity and amorphous phase fraction (>80%), which is impossible to achieve with other research methods.

## 3. Piezoelectric Photoacoustic Detection

In direct PA methods, the acoustic perturbation formed directly in the test sample is registered. Such methods are usually sensitive not only to the thermophysical and optical properties but also to the elastic properties of the studied sample. In addition, these methods are more sensitive and operate in a wider frequency range compared to the indirect PA methods described above. One of the main methods of recording an informative response is the piezoelectric detection technique. This method is quite simple for its practical implementation, as there is no need to place the sample in a closed volume of the gas cell.

As mentioned above, Rosenzweig [23] presented a one-dimensional approach for the description of the PA signal’s formation in the case of the gas–microphone technique. The proposed model can also be adopted for the study of the formation of temperature fluctuations during the piezoelectric approach. Jackson and Amer [42] developed a more precise three-dimensional theory of the piezoelectric detection technique. The theory can be applied to the simulation of the PA signal’s generation in solids with a wide range of parameters describing the thermophysical and optical properties. Blonskij [43] presented the piezoelectric theory to simplify the applications of the approach. The model was successfully applied for thermal diffusivity measurements of metals. Isaiev et al. [44] expanded the model for the simulation of the PA signal with piezoelectric detection for multilayered systems. In these systems, they used the buffer layer for the redistribution of thermoelastic force moments arising in the studied system. The model is based on the introduction of Green’s function formalism in the framework of Kirchhoff–Love theory. It was shown that the elastic properties and the width of this layer play an important role in ensuring better control of the contribution of different regions of the studied system to the detected voltage on the electrodes of a piezoelectric transducer. An excellent agreement between the simulations and experimental results was obtained for a heterogeneous porous silicon–bulk Si substrate structure. Fitting the experimental curves with the proposed model reveals the evaluation of the thermal conductivity of the mesoporous silicon with different porosity values. Let us consider the stages of some theoretical development of the piezoelectric method as an effective tool for studying various materials in detail.

### 3.1. Photoacoustic Signal Formation in Solids with the Piezoelectric Technique

**Figure 7:**
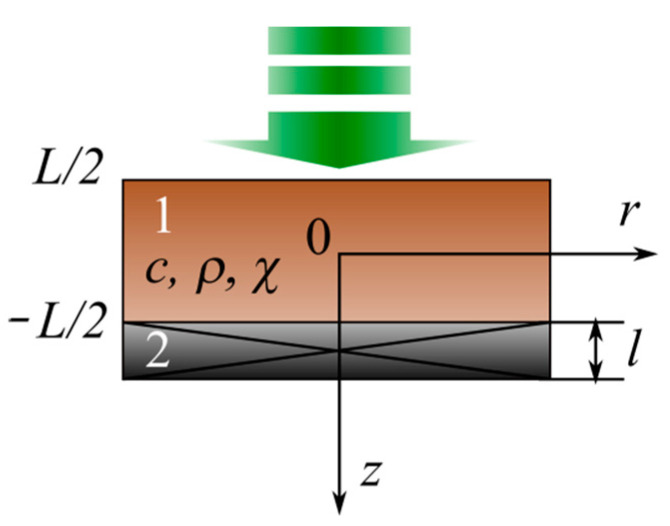
Schematic representation of the structure used to study the parameters of a photoacoustic response in piezoelectric registration.

Let us start by considering the approach to the study of the thermal conductivity of solids proposed in [43]. The authors considered a cylindrical sample with a thickness *L*, a radius *r,* thermal conductivity *χ*, a density *ρ*, and specific heat *c*. The upper side of the structure *z = L/2* was periodically illuminated by modulated radiation (see Figure 7), and the piezoelectric transducer was attached to the lower part of the sample (*z = −L/2*).

The spatial distribution of the variable component of temperature in the sample can also be obtained under the framework of the thermo-wave approach. Specifically, the heat transfer equation for the variable temperature component in three dimensions can be written as follows:

(19) $$\nabla^{2}T\left(r,\theta ,z\right)-\frac{1}{\alpha }\frac{dT\left(r,\theta ,z\right)}{dt}=-\frac{Q\left(r,\theta ,z\right)}{\chi }e^{-i\omega t},$$

where *Q*(*r, θ, z*) is the bulk density of heat sources and *ω* is the cyclic modulation frequency of the excitation radiation.

The propagation of the heat wave in the sample causes elastic stresses due to thermal expansion. The corresponding displacement of the sample (1 in Figure 7) creates a potential difference in the piezoelectric transducer.

To solve the problem of thermoelasticity, the theory of thin plates is used and it is assumed that the radius of the sample plate is much larger than its thickness. Considering the relationship between deformation and stresses and the boundary conditions on the sides of the sample, the expression for the sum of stresses in two directions, averaged over the area of the sample $\langle \varepsilon_{r}+\varepsilon_{\theta }\rangle ,$ can be written as follows:

(20) $$\langle \varepsilon_{r}+\varepsilon_{\theta }\rangle =2\pi \int_{0}^{R}\left(\varepsilon_{r}+\varepsilon_{\theta }\right)rdr=2\alpha_{T}\left(\langle T_{0}\rangle +z\langle \tau \rangle \right),$$

(21) $$\langle T_{0}\rangle =\frac{1}{L}\int_{-L/2}^{L/2}\langle T\left(r,z\right)\rangle dz,\;\langle \tau \rangle =\frac{12}{L^{3}}\int_{-L/2}^{L/2}\langle T\left(r,z\right)\rangle zdz,$$

where *ε_r_* and *ε_θ_* are the deformations in directions *r* and *θ,* respectively, and *α_T_* is the thermal expansion coefficient of the sample.

With strong optical absorption, the voltage *V* on the covers of the piezoelectric transducer can be recorded as follows:

(22) $$V=\frac{eL}{\varepsilon S}\langle \varepsilon_{r}\rangle +\langle \varepsilon_{\theta }\rangle =-\frac{2P\alpha_{T}}{\chi L\sigma^{2}}\left(1+\frac{3\left(1-\mathrm{ch}\left(\sigma L\right)\right)}{\sigma^{2}L\mathrm{sh}\;\left(\sigma L\right)}\right),$$

(23) $$P=2\pi Ib^{2}\frac{el}{\varepsilon S\;},$$

where *e* is the piezoelectric constant, *ε* is the dielectric constant, *S* is the surface area of the piezoelectric transducer, $\sigma =\sqrt{i\omega /D}$), *D* is the thermal diffusivity, *I* is the intensity of the incident radiation, *b* is the radius of the beam, and *l* is the thickness of the piezoelectric transducer.

Equation (22) describes the dependence of the voltage amplitude (phase) on the capacitor plates in the modulation frequency of the excitation radiation. Note that despite the relative simplicity, the results of this model are in good agreement with the results described in [42] and with the experimental ones.

Note that although the adiabatic approximation (the absence of heat outflow from the studied system) was used to calculate the thermal fields in this model, it must be taken into account [45] that the heat outflow from the surface of the material does not significantly affect the obtained results [46].

A disadvantage of the described approaches is the approximation of the thin piezoelectric transducer, which was used to describe the parameters of the PA response. In this approximation, it is assumed that its thickness is much smaller than the thickness of the test sample. Consequently, we consider the case where the sample oscillates freely and the piezoelectric transducer does not affect such oscillations. This assumption is particularly incorrect in cases when the thickness of the piezoceramic layer is proportional to or greater than the thickness of the sample. Therefore, it is problematic to use this approach for the analysis of experimental data in the study of inhomogeneous semiconductor structures, such as structures with modified properties of the surface layer and porous semiconductors. In this case, the ratio of the geometric dimensions of the system piezoelectric transducer–sample significantly affects the formation of the PA response [47].

The theoretical model of the PA response’s formation in a two-layer structure was developed in [48]. Here, the authors experimentally studied the effect of the piezoelectric transducer’s presence on the oscillations of the sample under the action of periodically modulated radiation. The proposed approach expanded the limits of the practical application of the PA method with piezoelectric detection in the material study, in particular, of thick samples [49] or non-rigid materials (for example, organic semiconductors [50]) and biological tissues [51].

Isaiev et al. [44] developed another model for describing the PA signal’s formation in heterogeneous multilayer structures under the framework of quasi-stationary approximation. The model is adopted for thermal conductivity measurements of mesoporous silicon layers etched on the bulk silicon substrates.

### 3.2. Experimental Setup of PA with a Piezoelectric Registration Technique

**Figure 8:**
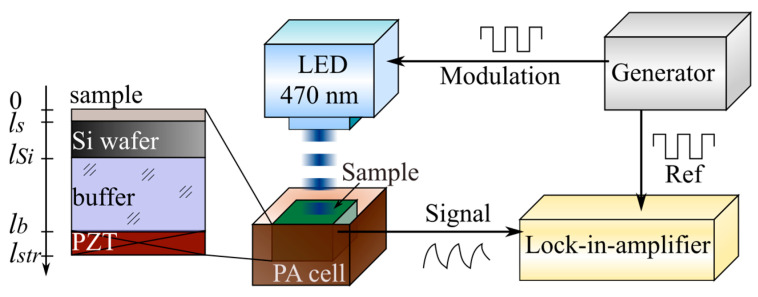
Sketch view of the experimental photoacoustic setup with piezoelectric detection.

A schematic representation of the experimental setup with a piezoelectric registration system is shown in Figure 8.

To perform a theoretical analysis of photoacoustic signal formation, the calculation of the photoinduced temperature profiles in a multilayered system is required. This can be achieved by solving the thermal diffusive equation for the considered structure:

(24) $$\frac{d^{2}\theta }{dz^{2}}-\frac{i\omega c_{S}\rho_{S}\theta }{\chi_{S}}=-\frac{I\left(1-R\right)\alpha_{S}}{\chi_{S}}e^{-\alpha_{S}z},\;0<z<l_{S},$$

(25) $$\frac{d^{2}\theta }{dz^{2}}-\frac{i\omega c_{Si}\rho_{Si}\theta }{\chi_{Si}}=-\frac{I\left(1-R\right)\alpha_{Si}}{\chi_{Si}}e^{-\alpha_{S}l_{S}}e^{-\alpha_{Si}\left(z-l_{S}\right)},\;l_{S}<z<l_{Si},\;$$

where *θ* is the temperature; *ω* is the cyclic frequency; *c*, *ρ*, and *χ* are the specific heat, density, and thermal conductivity of the sample and silicon substrate, respectively; *I* is the intensity of the exciting radiation; *R* is the reflection coefficient of radiation from the surface of the test sample; *α* is the optical absorption coefficient; and *z* is the depth of penetration of temperature perturbation. The S and Si indices are used to denote the parameters corresponding to the nanostructured sample and monocrystalline silicon, respectively.

The formation of the PA signal in a multilayer structure is considered under the framework of the quasi-stationary approximation and the rigid normal assumption. In this case, the spatial distribution of thermoelastic stresses *σ*(*z*) occurring in the system can be represented as follows:

(26) $$\sigma \left(z\right)=\frac{E\left(z\right)}{1-\nu \left(z\right)}\int_{0}^{l_{si}}G\left(z,s\right)\sigma_{th}\left(s\right)ds$$

where

(27) $$G\left(z,s\right)=\frac{m_{2}-zm_{1}-s\left(m_{1}-m_{0}z\right)}{m_{0}m_{2}-m_{1}^{2}},$$

(28) $$m_{n}=\int_{0}^{l_{str}}\frac{E\left(z\right)}{1-\nu \left(z\right)}z^{n}dz,$$

where *E*(*z*) and *ν*(*z*) are Young’s modulus and Poisson’s ratio, respectively; *G*(*z*,*s*) is Green’s function of the studied structure; $\sigma_{th}\left(s\right)$ is the source of stress; *s* is the point of application of the delta function; $l_{Si}$ is the thickness of the silicon; and $l_{s}$ is the total thickness.

In this case, the source of stresses $\sigma_{th}\left(z,\omega \right)$ is a thermoelastic force arising due to photothermal heating:

(29) $$\sigma_{th}\left(z,\omega \right)=\frac{\alpha_{T}\left(z\right)E\left(z\right)\theta \left(z,\;\omega \right)}{1-\nu \left(z\right)},$$

where $\alpha_{T}\left(z\right)$ is the distribution of the thermal expansion coefficient in the material and θ(z, ω) is the distribution of the variable temperature component on the frequency *ω*.

The resulting voltage induced on the electrodes of the piezoelectric transducer is:

(30) $$U\left(\omega \right)\;\sim \int_{l_{buf}}^{l_{str}}\sigma \left(z,\omega \right)dz$$

The described theoretical approach was used to analyze the experimentally obtained amplitude–frequency and phase–frequency characteristics. The developed model is in excellent agreement with the experimental results. Thus, solving the inverse problem by fitting the experimental curves with the theoretical curves enables precise measurements of the thermal conductivity of the nanostructured porous silicon layer.

The shape of the PA signal was additionally analyzed. The recorded time-resolved voltage was simulated by the following expression:

(31) $$U\left(t\right)=\sum_{n=-\infty }^{\infty }U\left(\omega_{n}\right)q_{n}e^{i\omega_{n}t}$$

where $U\left(\omega_{n}\right)$ are the Fourier components of the voltage obtained from Equation (30), $q_{n}$ are the Fourier components of the excitation signal, and $\omega_{n}$ = 2π$f_{n}$ are the cyclical frequencies. It was shown that the PA signal shapes are dependent on the geometrical and elasticity parameters, as well as on the thermophysical properties of the material.

### 3.3. Application of PA in Piezoelectric Detection Configuration

#### 3.3.1. Thermal Property Characterization of Nanostructured Materials with the Photoacoustic Piezoelectric Technique

Various heterogeneous systems are widely studied and used in numerous scientific fields. Some examples of these systems include epitaxially grown multilayers, porous materials, and composite systems. The piezoelectric detection technique can be successfully applied for the study of such structures. As mentioned above, this method allows us to obtain information about the thermal properties of the material.

First, let us consider the study of the thermal properties of inhomogeneous nanostructured systems based on silicon. The model presented in [44] has been successfully applied to the thermal conductivity studies of porous silicon. It was found that the thermal conductivity is equal to 2.1 and 0.7 W/m·K for porous silicon (PS) layers with porosities of 48% and 65%, respectively.

Later, the model of the formation of a photoacoustic signal with piezoelectric recording was modified for describing nanostructured objects such as silicon nanowire (SiNWs) arrays, which allowed us to evaluate their thermal conductivity [52]. The experimental data of the amplitude–frequency and phase–frequency dependencies of the photoacoustic response from the layered structure containing SiNW arrays were detected by a piezoelectric transducer in a frequency range from 10 to 20 kHz. The stresses in tangential directions inside the studied sample have been neglected to approximate the experimental data. The approximation is valid since the layer of the studied material consists of separated nanowires without mechanical contacts. Therefore, the thermoelastic component of the photoacoustic signal from the layer with SiNW arrays is absent. The thermoelastic component of the photoacoustic signal from the silicon substrate prevails over the entire frequency range. All the values evaluated in the paper are well correlated with the values of similar Si nanomaterials reported earlier in the literature. In particular, due to the influence of fractal-like fine morphological features of the porous silicon network, its thermal conductivities were found to be systematically lower compared to the thermal conductivity values of the quasi-straight silicon nanowires.

An approach using the simultaneous evaluation of thermal conductivity and the heat capacity of nanostructured silicon-based materials was proposed in [53]. The experiments were carried out in the cases of the presence and absence of a covering liquid layer (water) on top of the studied structure. The presence of the layer leads to the redistribution of the deposited energy at the surface of the nanostructured sample. The heat fluxes at the nanostructured solid–fluid interface should be equal due to energy conservation in the system:

$$\chi_{l}{\frac{\partial T}{\partial z}|}_{z=0-0}=\chi_{s}{\frac{\partial T}{\partial z}|}_{z=0+0}$$

where $\chi_{l\;}$ and $\chi_{s\;}$ are the thermal conductivities of the covering liquid and the sample under consideration, respectively. The evaluation of the thermal properties of PS samples with different porosities and SiNW arrays with different thicknesses was performed by minimizing the difference between experimental amplitude–frequency characteristics and the simulated ones. In this case, thermal conductivity and heat capacity were taken as varied parameters. It was shown that the heat capacity of the nanowire arrays decreases as the layer thickness increases.

Another example of the application of the piezoelectric PA method is the study of the local stresses arising in the systems under elastic loads. As an example, the study of the PA response near Vickers-indented areas in ceramics demonstrated the significant dependence of acoustic signals on internal and external stresses at the tips of near-surface cracks. The PA signal’s formation inside Vickers indents in metals showed the same behavior in [54]. PA imaging close to the indented areas was obtained by scanning the sample’s surface using the laser beam. The presented experimental results for nano-nickel and steel samples demonstrate the following feature of the PA signal’s formation inside the Vickers indentation zone: strong changes in the signal take place at stress concentrators.

The photoacoustic method with piezoelectric signal registration is also widely used to study the thermal properties of various composite systems. Thus, in [55], a photoacoustic piezoelectric detection technique was developed to study two-layered materials and evaluate thermal diffusivity of a phosphor/PVA light-emitting composite material. The dependencies of the phase of the PA signal on the modulation frequency were experimentally measured. The thermal diffusivity coefficient and the ratio of elastic parameters were evaluated by the fitting. The calculated thermal diffusivity of phosphor/PVA light-emitting composite material was 3.95 mm^2^/s, and the ratio of elastic parameters was 0.04.

The thermal diffusivity of fiber-reinforced composites was studied by a PA piezoelectric approach based on a simplified thermoelastic theory in [56]. The studied material was the composite Al_2_O_3_x/SAE321 with short Al_2_O_3x_ fibers with a length of ~200 µm and a diameter of ~10 µm and a randomly reinforced Al alloy matrix (SAE321). This approach offers the possibility of obtaining average effective thermal properties contrary to other photothermal methods.

Composite systems based on porous matrices filled with liquid are widely used in biology, chemistry, and medicine. Therefore, from an application point of view, it is important to know the physical properties of these structures. The investigation of such properties allows us to obtain information about properties of the porous matrix, the behavior of the liquids inside the pores, as well as the whole composite system. Andrusenko et al. [57] performed an experimental and theoretical study of the photoacoustic effect in a porous silicon–liquid composite system. Particularly, the effect of thermally induced pressures of the liquid inside the pores on the shape of the photoacoustic signal was analyzed in detail. In [58], a model of photoacoustic response formation in composite systems based on porous silicon matrices with different morphologies under their irradiation with periodically modulated radiation was proposed. The developed model considers changes in thermophysical and elastic properties due to fluid incorporation.

#### 3.3.2. Characterization of Colloids, Liquid Food, and Nanofluids Using the Piezoelectric Photoacoustic Technique

Many properties of fluids (such as colloids, nanofluids, liquid food, and magnetic fluids) can be exploited in a wide range of applications, for example, in machining processes, solar energy, theranostics, and medicine [59,60,61,62,63]. Studying the physical properties of various types of liquids is important for the efficient development of their various multidisciplinary applications. If the energy of the used fluence is below a threshold value, the regime is called thermoelastic. The laser–matter interaction produces acoustic waves that can be detected by a piezoelectric transducer.

Because PA response parameters often depend only on the absorption portion of laser irradiation, the PA method with the piezoelectric detection principle can be widely used to study the optical properties of highly scattered media, such as various colloids. Specifically, when the laser pulse duration is much less than the propagation time of an acoustic wave over the heated region, the PA signal follows the spatial distribution of optical absorbance in the studied medium. Therefore, the time profile of the registered PA signal is determined by the light absorption and scattering coefficients. The amplitude of the PA signal also depends on the absorbed energy, as was mentioned above. It offers the possibility of decomposing the impact of the absorption and scattering on the light propagation in transparent media. Thus, the photoacoustic signal measurement of colloids obtained by laser ablation in liquids can be used to study the changes in their optical properties [64].

Magnetic fluids, which are a type of stable colloidal liquid, possessing both the fluidity of liquids and the magnetic properties of colloidal magnetic solids, have attracted interest due to their prospective applications [65,66,67,68,69,70]. Authors of Refs. [68,69] performed a laser photoacoustic study of the structural inhomogeneity of a magnetic fluid by the reconstruction of the spatial distribution of the light extinction coefficient.

In [71], the feasibility of an assembled piezoelectric-based PA system for label-free fluid flow monitoring was discussed. The measured PA signal detected by a piezoelectric transducer was processed to determine the required phase value, which was found to correlate linearly with the fluid flow status.

Additionally, the possibilities of application of PA approaches in the assessment of the thermal conductivity and thermal diffusivity of nanofluids were discussed in [1].

The application of the PA piezoelectric approach in the evaluation of thermal properties of nanofluids was demonstrated in [72], where the dependence of thermal conductivity on the concentration level of the carbon fluorooxide (CFO) [73] mesoparticles was investigated. Different basic liquids were considered, such as polyethylene glycol (PEG), oil, and water. The amplitude–frequency characteristics of the CFO-based nanofluids with various concentrations of particles expressed in terms of volume fraction were measured. It was shown that variation in the amplitude–frequency slopes in the double logarithmic scale is related to different physical properties of the considered nanofluids. Phase–frequency dependencies give another marker for following a further delay of thermal perturbation propagation through the studied liquid. Such features of the curve’s behavior allow for making a reliable estimation of the thermal conductivity used as a single fitting parameter in the simulations. The obtained experimental values of thermal conductivity are in the range from 0.1 to 1 W/m·K.

In [74], the authors described the development, implementation, and evaluation of the Nutrilyzer photoacoustic sensing system, which is able to characterize various types of liquid food. The device uses an array of LEDs, two piezoelectric sensors, and an ARM microcontroller unit to measure the PA response excited by the transmitted and scattered light beam in the solution located in a cuvette. The unique spectral signature captured by these two piezoelectric sensors across all the 16 LEDs in the array enables us to capture the optical transmission and scattering properties, which eventually offers the possibility of detecting nutrients and adulterants in liquid food.

Kishor et al. [75] developed a novel integrated surface-acoustic-waves-based photoacoustic (SAW-PA) sensor platform. The platform enables the evaluation of the optical and mechanical properties of fluids flowing through a microchannel. In the setup, the longitudinal acoustic wave was excited and then it was converted into a Rayleigh interfacial wave at the liquid–LiNbO_3_ interface. The piezoelectric LiNbO_3_ substrate was used for the registration of the acoustical response. In such a configuration, it was possible to measure the liquid concentration in a microchannel with the use of two standard dye solutions. Moreover, the impact of viscosity on the SAW-PA signal was identified, which also offers the possibility of studying the rheological properties of the liquid. The developed system was also used for the investigation of the SAW-PA response of the liquids containing plasmonic nanoparticles, such as spherical gold nanoparticles (AuNPs) and gold nanorods (AuNRs), of different sizes as a test system. The PA signal generated by AuNPs shows a nonlinear tendency with the size of nanoparticles, which improves the sensitivity. Further, the detection of the sensitive longitudinal plasmon resonance mode of AuNRs at 1064 nm demonstrates the possibility of using the platform for biosensing.

## 4. Physical Principles of Photoacoustic Imaging

The discovery of the PA effect also stimulated the development of various approaches for the visualization of the object, which was later called photoacoustic imaging (PAI) [76]. The first low-resolution and contrast images were focused on obtaining information from various inorganic materials, such as metallic and semiconducting structures [77]. The limitations of this technique were due to the inherent drawbacks of the systems related to focusing monochromatic optical radiation and positioning the beam on the sample. Subsequent technical progress has played an important role in the development of this technique. The modern PAI technique reconstructs the simultaneous real-time imaging of surface and subsurface structures with high contrast and micron-scale spatial resolution [78,79,80]. In this section, we introduce technical advances and the applicability of the photoacoustic technique in microscale imaging. In addition, we briefly emphasize the role of nanostructures in improving the visualization of objects using the PAI technique.

PAI methods have several advantages over optical microscopy, namely (i) they provide a deeper imaging depth (up to hundreds of micrometers for PAM and up to several centimeters for PAT), (ii) they do not need optical sectioning to provide a 3D PA image, and (iii) they can analyze the samples that are too absorbing, too scattering, or too transparent for optical microscopy methods.

According to the configurations of optical excitation sources and PA signal detection methods, the implementations of PAI can be classified into two broad categories [81]:

(a) Photoacoustic microscopy (PAM), a common low-cost method where a focused modulated laser beam is used to achieve high-spatial-resolution imaging at small depths;
(b) Photoacoustic tomography (PAT), a hybrid imaging technique that combines optical excitation and acoustic detection to realize imaging with relatively deep penetration in the studied object.

### 4.1. Photoacoustic Microscopy: Areas of Application, Advantages, and Drawbacks

At present, PAM is known to be one of the most powerful techniques for the non-destructive testing of surface flaws and subsurface inhomogeneities of the studied objects. This concept is provided by using a non-stationary laser with the ability to move its beam through the sample’s surface and detect the PA response This is achieved, for example, by moving the sample itself using stepper motors or manipulating the beam using acousto-optic deflectors. According to the process of PA signal registration, the most commonly used PAM detection schemes are the gas–microphone technique and a method that uses a piezoelectric transducer.

In the case of the low-frequency modulation of the laser (from Hz to kHz), the PA signal depends on (a) the optical absorption of the sample, (b) the generation and propagation of thermal waves, and (c) elastic waves. Thus, a number of factors contribute to the characterization of a sample based on the analysis of its PA response.

A combination of PAM with other methods can provide aggregate information about the target sample, complementing different types of imaging [81,82,83]. As scanning modes, it is possible to use various methods for recording the PA response, methods such as optical images for the transmission or reflection of light, the photoelectric (PE) method, etc. The choice of the scanning mode depends on the possibility of adapting the system to the structural features of the sample, as well as the research objectives.

In particular, PAM, complemented by images in the PE mode of semiconductor materials, can visualize any irregularities that are elastically stressed regions, disturb the propagation of the thermal diffusion wave, and/or affect the optical absorption coefficient that is difficult to see with an optical microscope (for example, subsurface voids, microcracks, the crystallographic defects of substrates, and the delamination of layered materials) [83]. Furthermore, the use of confocal microscopy coupled with the PA approach offers the possibility of estimating the size of the nanoparticle’s agglomeration [84].

**Figure 9:**
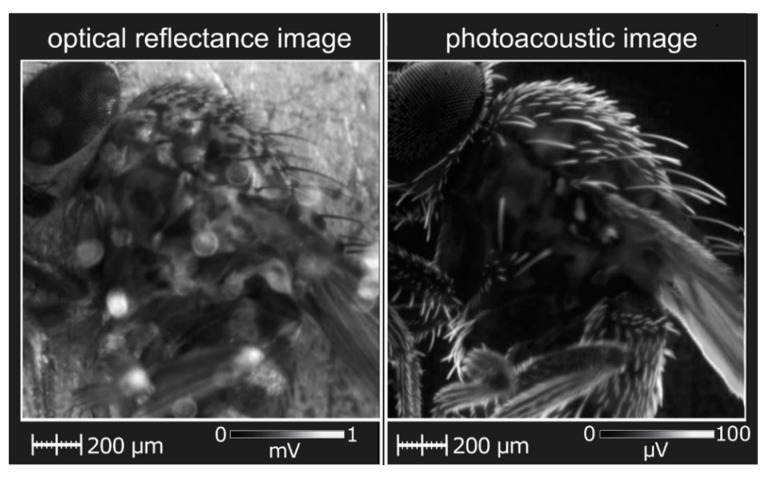
PAT images of a Drosophila fly obtained by a bi-modal system in the reflected light and PA mode.

The use of a dual-mode PAM/optical imaging system has been implemented, for example, for visualizing organic objects, such as the Drosophila fly (see Figure 9) [82]. The obtained PA images complement the images based on the reflected light and reveal the features of the microscopic anatomy of the insect that can be accurately seen only with high-precision and expensive devices such as SEM.

It should be noted that low-frequency PA microscopy, along with its advantages, also has several technical and engineering problems that need to be solved. Among them, motion artefacts (if a living object is used as a target), imaging field limitations, and low scanning speed play an important role. In addition, most modern PAM systems are applicable only for the visualization of small areas (up to several hundred square millimeters), limited both by instrumental factors (slow scanning speed, the capabilities of sensors for detecting the PA signal, etc.) and by the nature of photothermoacoustic processes (fast extinction of the heat wave in the sample, etc.).

To overcome motion artefacts from living objects under study, they are typically well-fixed and/or in anesthesia. However, the anesthetic or uncomfortable fixation effect may affect physiological functions, such as neuron activities and metabolism, contrary to normal conditions. These factors can give a false picture of the vital signs of the object during various kinds of research. The issue of analyzing conscious living organisms using PAM methods at this stage has not been resolved and requires significant engineering solutions (the miniaturization of a cell, its incorporation into an organism, a significant reduction in scanning time, etc.).

### 4.2. Adaptation of Photoacoustic Tomography for Preclinical and Clinical Applications

Photoacoustic tomography (PAT) characterizes the optical properties of a studied sample from acoustic signals excited by the absorption of modulated electromagnetic radiation. The imaging of optical absorption reveals important information about the internal environment in phantom or biological tissues [85,86]. In general, the PA response is excited by pulsed laser radiation with a pulse duration of ~10 ns in the visible and near-infrared spectral range, and the acoustic waves, which appear after the absorption of radiation and are caused by the thermoelastic expansion of the media, are detected by various broadband sensors, analyzed in detail in [87].

In PAT, images are reconstructed by applying appropriate tomographic algorithms to the acquired time-resolved signals for an accurate reconstruction of imaging. To create a high-quality image, several hundred to tens of thousands of detector positions are usually used. To achieve this high number of sensors, several approaches could be applied. Dean-Ben et al. [88] developed a volumetric multispectral optoacoustic tomography platform for imaging a neural activation deep in scattering brains. Such a platform contains 512 elements, which allow us to obtain single-shot, 3D tomographic images. The reconstruction of an image by applying a 3D algorithm can be performed by a curved array rotating relative to the studied object, emulating a spherical array with thousands of virtual sensor points [89]. A PAT image can be reconstructed by a series of 2D sectional images generated by a ring-shaped array that encloses the studied object and is then translated along the ring axis [90,91]. Additionally, one can independently analyze each 2D slice. The latter allows for more precision, considering the time-dependent processes in a slice. For instance, Paltauf et al. presented an array of extended, line-shaped detectors that generate 2D projections of the photoacoustic sources for photoacoustic tomography [92,93].

The possibility of the noncontact piezoelectric detection of photoacoustic signals in tissue phantoms was analyzed in [94] in detail. The authors studied human blood flow in a silicon rubber tube mimicking a blood vessel with an inner diameter of 4 mm. The photoacoustic time traces were recorded by the transducers, which were located at a 7.5 mm distance from the phantom interface, at different frequencies. It was shown that the sensitivity of the air-coupled ultrasound transducers is sufficient to detect PA signals generated by an artificial blood vessel. The latter allows for the integration of PA noncontact piezoelectric detection simultaneously with the X-ray mammographic screening procedure.

Gao et al. [95] applied the piezoelectric photoacoustic method based on a simplified thermoelastic theory to evaluate the thermal diffusivities of some biological tissues. Particularly, the thermal diffusivities of porcine tissues (skin, fat, muscle, heart, liver, and kidney) with different preparation conditions were assessed. They showed that the thermal diffusivities of the fresh tissues are higher in comparison with those of the dried and specially prepared tissues. This may occur because evaporating the tissues increases the discontinuity in the tissues and fixing the proteins and fats of the tissues increases the thermal resistance. Both these effects decreased the thermal diffusivities of the tissues. The obtained results show that the photoacoustic method with piezoelectric detection is effective for evaluating the effective thermal diffusivities of the tissues with micro-inhomogeneities.

Kolkman et al. [96] demonstrated the possibility of the application of photoacoustic imaging (PAI) in the detection of blood vessels inside the tissue in a non-invasive way. The biological tissue is irradiated by a short light pulse, which is partly absorbed by the particles, such as red blood cells. The resulting acoustic wave propagates through the tissue and can be detected at the surface by a piezoelectric transducer.

The photoacoustic technique with piezoelectric detection (by using a fast PZT-ceramic acoustic transducer) also allowed for the in vitro and in vivo study of individual cells and nanoparticles in [97]. The latter offers the possibility for the diagnosis of breast cancer labeled with gold nanoparticles. Together with further hyperthermia treatment, this allows us to develop an efficient theranostic approach for breast cancer.

Among other areas in therapy, the analysis of the glucose level in blood or interstitial fluid is crucial for diabetic diagnosis. Kottmann et al. studied glucose concentration in the human epidermis by the PA method for the first time [98,99]. They used an external-cavity quantum-cascade laser and a gas–microphone method for the detection of acoustic signals described above. The detection limits of glucose concentration were obtained in the physiological range of 30–500 mg/dL. Nevertheless, for monitoring the glucose levels non-invasively, some improvements are necessary. In [100], a tunable pulsed laser for a photoacoustic method with piezoelectric detection was used for measurements of glucose concentration. However, a significant disadvantage of such studies is the need to use expensive microphones and quantum cascade lasers.

Bayrakli et al. [101] developed a PA sensor with an inexpensive piezoelectric film transducer as an acoustic detection module and an amplitude-stabilized external cavity diode laser source. A wavelength equal to 1050 nm of the source was chosen for glucose concentration analysis. This sensor offers the possibility of achieving a detection limit of 900 mg/dL. In [102], the theory of liquid photoacoustic resonance was developed. The relation between signal characteristics and the concentration of the glucose solution was obtained. The experimental results show that a liquid’s photoacoustic resonance can enhance the signal and the resolution thus achieved is 20 mg/dL. Therefore, the proposed method overcomes the issue of low sensitivity and inaccurate detection in nonresonant cases.

## 5. Physical Principles of Photoacoustic FT-IR Spectroscopy

Fourier transform infrared spectroscopy (FT-IR) is currently one of the widely used systems for the analysis and identification of functional groups in solids, liquids, and gases. FT-IR spectra can be used for sensing chemical substances and bonds.

The main problem with the FT-IR analysis of semi-solid and solid materials is that almost all of these materials are too opaque for the direct analysis of optical transmission in the mid-infrared region of the spectrum [103]. Another significant problem is the scattering of the IR light, which can significantly perturb the spectra from nanostructured materials and biological tissue.

From this point of view, the most versatile solution to the problems of opacity and scattering in the mid-infrared range is Fourier transform photoacoustic infrared spectroscopy (PA FT-IR).

The PA FT-IR method is based on the photoacoustic effect. Generally, the illumination of samples in different aggregate states with electromagnetic radiation is carried out in the IR wavelength range. The absorption of this radiation causes local changes in temperature inside the sample, leading to its thermal expansion. These processes create acoustic waves in the near-surface region of the sample, isolated from the environment, which is captured by a highly sensitive microphone, the vibration amplitude of which is measured using a laser interferometer [104]. In the current section, we will consider the application of the PAS in the characterization of solid and liquid samples with nanostructured phases.

### 5.1. Gas-Phase Photoacoustic FT-IR Spectroscopy

Gas–microphone configuration is the most used configuration for PA FT-IR measurements. There are several solutions presented in stock that are compatible with different FT-IRs [17].

**Figure 10:**
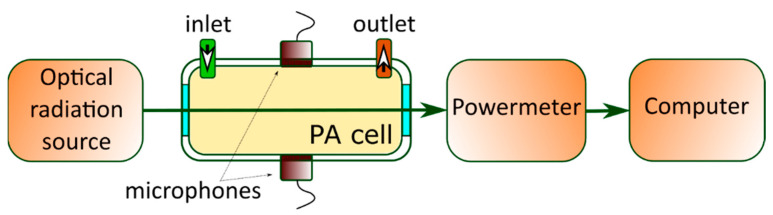
Schematic sketch view of the PAS setup’s configuration.

As described above, in PAS, the absorbing gas medium enclosed in a specially designed PA cell (see Figure 10) [105] absorbs laser radiation at the desired frequency. The energy absorbed by the gas is translated into gas pressure variations through non-radiative relaxation processes, which cause acoustic waves inside the PA cell to be detected by microphones. In the resonance, the PA configuration’s frequency of modulation is chosen from standing waves in the considered cell [106].

**Figure 11:**
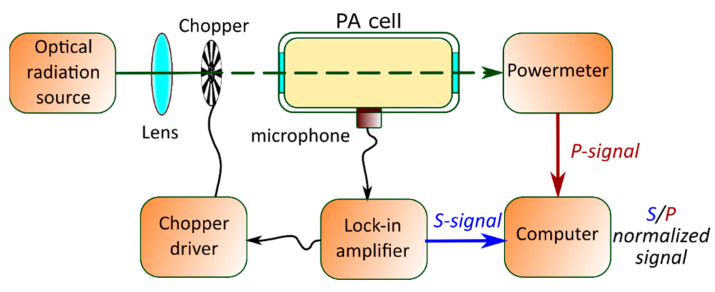
Typical setup for PAS measurements with resonance PA cells.

The respective frequency of modulation can be achieved by the interruption of the continuous wave laser radiation by a mechanical chopper operating at an acoustic resonance frequency of the PA cell. It is then focused by a lens and directed through the resonant PA cell. In such a configuration (see Figure 11), the transmitted laser power (signal P) can also be detected with a power meter. Moreover, the PA signal (S) can be analyzed with a lock-in amplifier and the normalized absorption can then be deduced as the S/P ratio.

A typical application of PA spectroscopy is the detection of atmospheric CH_4_, CO_2_ [107] ppm, NO_2_ [108], and N_2_O, and sensitivity in the range of a few parts per billion could be achieved [109]. For further insight regarding PA gas sensing, one can refer to the recent reviews [2,105,106].

### 5.2. Solid- and Liquid-Phase Photoacoustic FT-IR Spectroscopy

The physical principle of PA-based IR spectroscopy is similar to the one described in previous sections. In this case, the gas–microphone and piezoelectric configurations are often used for signal detections [1]. However, some specificity of the signal formation should be considered to distinguish the optical properties of the considered media with good precision.

**Figure 12:**
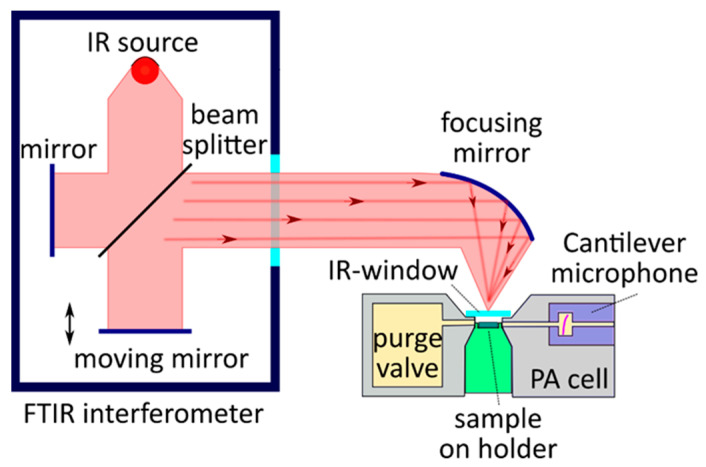
Schematic view of a typical photoacoustic Fourier transform infrared (PA FT-IR) setup. Reprinted with permission from ref. [17]. Copyright 2021 Gasera Ltd. Oy.

A sketch of an IR spectrometer with PA cells for the analysis of solid and liquid samples is presented in Figure 12 [17]. This setup consists of an IR spectrometer, a focusing system, and a PA cell with a sample. IR radiation from the spectrometer is focused on the sample’s surface and causes local heating of the sample. The magnitude of the heating depends on the absorption at the specific wavelength. Thus, scattering phenomena do not directly influence the registered signal. One can associate the absorbed portion of light at a specific depth with the surface temperature based on thermal problems similar to the ones described in Section 2.

At the moment, condenser microphones remain the most common sensors for measuring the PA response from a sample during its analysis by photoacoustic spectroscopy [110]. The alternative piezoelectric transducer is not so widely used in such types of systems due to the significant mismatching of the acoustical impedance between the piezoelectric material and the surrounding gas.

A cantilever pressure sensor is another promising sensor type that has begun to be used in modern PA FT-IR spectroscopy [111,112,113]. The main advantages of the cantilever over other types of sensors are its wide dynamic range of motion and low string constant, which is several orders of magnitude less than for membranes of other sensors. The movement of the cantilever can be tens of micrometers, without any limiting and non-linear effects. To avoid damping due to the probe and to maintain a wide dynamic range, it is necessary to provide an interferometric measurement of the cantilever tip’s motion [114].

**Figure 13:**
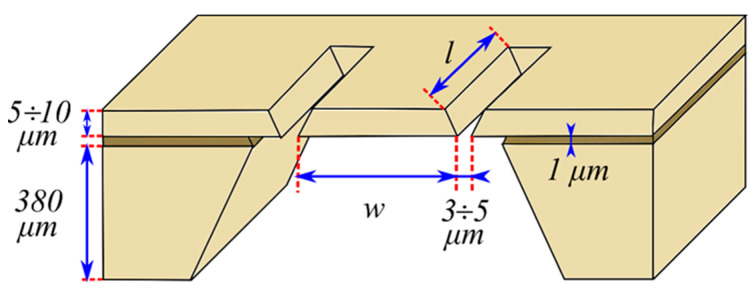
Silicon cantilever sensor. Typical dimensions for width *w* and length *l* are a few millimeters [116].

An example of the widespread use of a cantilever pressure sensor with significantly improved sensitivity for PAS applications is the sensor developed and patented by Gasera. This cantilever is fabricated from silicon using a controlled double-sided micro-etching process for silicon-on-insulator (SOI) wafers [115]. The movement of this sensor, caused by the differential pressure over the console, is similar to the movement of a saloon door. The cantilever itself is usually a few microns thick and rests on thicker frames, the thickness of which reaches several hundred microns. The cantilever is surrounded on three sides by a micrometric gap that separates it from the frame structure. A schematic drawing of a cantilever’s structure and its typical dimensions is shown in Figure 13 [116].

The presented solution for the design of a cantilever is capable of providing ultra-high-sensitivity PA spectra in a few seconds, which is difficult to achieve with alternative available photoacoustic sensors. In addition, in this system, it is sufficient to use ambient air as a carrier gas to obtain the signal-to-noise ratio, since this still gives a better result in comparison with other PA detectors that use helium as a carrier gas [117].

For example, in [104], the results of the application of FT-IR spectroscopy are presented, where a cantilever was used as a sensor to determine the organic composition of mixed linker metal–organic frameworks. It was shown that this setup makes it possible to distinguish between azido and amino linkers on three different MOF platforms with a sensitivity that is 2 orders of magnitude higher than when using classical IR spectroscopy.

## 6. Conclusions

In this review, we generalized photoacoustic measurement methods for the characterization of thermal and optical properties of various nanomaterials. Such methods are extremely promising for the study of complex and widely used nanoscale media with heterogeneous nanostructured features. Specifically, a description of the photoacoustic approaches with gas–microphone and piezoelectric recording principles was presented in detail. The main advantages and disadvantages of both approaches were described and compared. Finally, the application of the photoacoustic approaches in PA imaging and infrared spectroscopy was reviewed.
